# Birth Prevalence of Homocystinuria in Central Europe: Frequency and Pathogenicity of Mutation c.1105C>T (p.R369C) in the Cystathionine Beta-Synthase Gene

**DOI:** 10.1016/j.jpeds.2008.09.015

**Published:** 2009-03

**Authors:** Miroslav Janošík, Jitka Sokolová, Bohumila Janošíková, Jakub Krijt, Veronika Klatovská, Viktor Kožich

**Affiliations:** Institute of Inherited Metabolic Disorders, Charles University in Prague—1st Faculty of Medicine, Prague, Czech Republic

**Keywords:** CBS, Cystathionine beta-synthase, CHO, Chinese hamster ovary, PAGE, Polyacrylamide gel electrophoresis, PBS, Phosphate buffered saline solution, SAM, S-adenosylmethionine, SAH, S-adenosylhomocysteine, SDS, Sodium dodecyl sulfate

## Abstract

**Objectives:**

To estimate the frequency of the cystathionine beta-synthase deficiency caused by c.1105C>T mutation in Central Europe compared to Norway, and to examine the pathogenicity of the corresponding p.R369C mutant enzyme.

**Study design:**

Mutation c.1105C>T was analyzed in 600 anonymous Czech newborn blood spots. Catalytic activity and quaternary structure of the p.R369C mutant was evaluated after expression in 2 cellular systems.

**Results:**

Population frequency of the c.1105C>T mutation was 0.005, predicting the birth prevalence of homocystinuria of 1:40 000, which increased to 1:15 500 in a model including 10 additional mutations. In *Escherichia coli* the p.R369C mutant misfolded, and its activity was severely reduced, and expression in Chinese hamster ovary cells enabled proper folding with activity decreased to 63% of the wild-type enzyme. This decreased activity was not due to impaired K_m_ for both substrates but resulted from V_max_ lowered to 55% of the normal cystathionine beta-synthase enzyme.

**Conclusions:**

The c.1105C>T (p.R369C) allele is common also in the Czech population. Although the p.R369C mutation impairs folding and decreases velocity of the enzymatic reaction, our data are congruent with rather mild clinical phenotype in homozygotes or compound heterozygotes carrying this mutation.

Homocystinuria caused by cystathionine beta synthase (CBS) deficiency is a rare autosomal recessive disorder of sulfur amino acid metabolism. The disease manifests in childhood by vascular, neurologic, and connective tissue abnormalities,^1,2^ although increasing numbers of mildly affected adults with only thromboembolism are reported.^3-6^

Various approaches have been used to examine the population frequency of homocystinuria, but the true incidence of the disease is unknown. Newborn screening, based on finding individuals with elevated blood methionine concentrations, has apparently been detected almost exclusively patients with severe pyridoxine nonresponsive form of disease^7^ and with a worldwide incidence of 1:65 000-1:900 000.^8^ However, estimates calculated from the heterozygote frequency for the most common mutation c.833T>C (p.I278T) in newborns suggested an incidence of at least one order of magnitude higher.^9-11^ In agreement with these findings Refsum et al^12^ reported even higher population frequency of another CBS mutation c.1105 C>T, which was present in 0.8% of CBS chromosomes among unselected Norwegian newborns. The calculated incidence of homocystinuria caused by 6 mutations including the c.1105 C>T may be thus unusually high with 1 patient with CBS deficiency expected in each of 6400 Norwegian newborns.^12^

The c.1105 C>T mutation is localized in a CpG dinucleotide in exon 10 and leads to replacement of arginine in position 369 of the CBS polypeptide by cysteine (p.R369C). The pathogenicity of this mutation is unclear. The c.1105 C>T was originally described in 3 unrelated pyridoxine-responsive patients of Norwegian,^14^ Dutch,^13^ and Anglo-Celtic^4^ origin, which strongly supports the notion of its pathogenicity (Table I). In contrast, Kim et al^14^ showed in a yeast system that expression of the p.R369C variant ensures normal to above-normal growth of CBS-deficient strain, suggesting none or at most a mild effect of p.R369C on the enzyme activity.

High frequency of the c.1105 C>T allele and its putative pathogenicity may have important epidemiologic consequences, such as for neonatal screening of homocystinuria. Our study was therefore aimed at (1) determining the frequency of this variant in another Caucasian population and (2) examining the pathogenicity of the p.R369C mutant by expression studies in prokaryotic and eukaryotic systems.

## Methods

### Samples

We used 300 anonymous peripheral blood spots from Prague and 300 archived umbilical cord blood samples from Brno representing Bohemian and Moravian region, respectively, from a study that was described previously.^10^ Genomic DNA was isolated with the QIAamp DNA Mini Kit (Qiagen, Valencia, California). This study was approved by the Ethics Committee of Charles University in Prague—First Faculty of Medicine.

### Genotyping

To detect the c.1105C>T alleles, we used polymerase chain reaction (PCR)-RFLP technique to amplify exon 10 from genomic DNA using specific primers—forward: 5′-CAgTgCCCACCCCAgCTCATTA-3′ and reverse: 5′-ggCCTCCTCCCCTCCCAgTTCT-3′ and Klentaq polymerase (GeneAge Technologies, Praha, Czech Republic).

Kim et al^14^ used *Hae*II for PCR-RFLP analysis of c.1105 C>T (p.R369C); however, the loss of restriction site in their assay may be also caused by the mutation c.1106 G>A (p.R369H). To explore the possibility that either c.1105 C>T or c.1106 G>A was present in Czech newborns, the PCR products were first digested with *Hha*I to detect these 2 mutant alleles. The *Hha*I-positive samples were further digested with *Cac8*I and *Dra*III to check for the presence of c.1105 C>T and c.1106 G>A mutation, respectively. The latter mutation was not detected in the studied cohort.

### Expression Constructs

Both the wild-type CBS and the mutant c.1105C>T constructs were derived from pHCS3 expression plasmid.^15^ The mutation was introduced into the wild-type expression plasmid with GeneTailor Site–directed mutagenesis kit (Invitrogen, Carlsbad, California) on the basis of manufacturer procedure. The sequences of mutagenic primers for c.1105 C>T were as follow—sense: 5′-gCTgCgTggTCATTCTgCCCgACTCagTgC-3′ and antisense: 5′-gCAgAATgACCACgCAgCACTggCCCTCCT-3′.

For the purpose of eukaryotic expression both the wild-type and c.1105 C>T containing CBS were cloned into pTRE2hyg vector (Clontech Laboratories, Mountain View, California). The presence of c.1105 C>T and the absence of additional mutations in the entire coding CBS sequence of the pHCS3 and pTRE2hyg constructs were verified by dideoxy sequencing with ALF sequencer (Amersham Pharmacia Biotech, Piscataway, New Jersey).

### Prokaryotic Expression

Both the wild-type and p.R369C CBS enzymes were expressed in DH5alpha cells (Gibco BRL, Carlsbad, California). The cells were grown at 37° C or 18° C, respectively, in Super Optimal Broth (SOB) media supplemented with ampicillin 100 μg/mL. After the OD_600_ of cultures reached ∼0.5, the cells were induced with isopropyl β-D-1-thiogalactopyranoside (final concentration 0.33 mmol/L) and grown for an additional 3 hours or overnight at 37° C or 18° C, respectively. The pKK vector without CBS insert was used as a negative control. Lysates from *Escherichia coli* cell pellets were prepared by sonication in Tris-Cl buffer with addition of Protease Inhibitor Cocktail suitable for use with bacterial cell extracts (Sigma Aldrich, St. Louis, Missouri).^15^ Concentration of protein in lysates was determined as described by Lowry et al^16^ with bovine serum albumin used as a standard. All expressions were done in triplicate, and the numbers shown in this article represent means.

### Eukaryotic Expression in CHO-K1 Cells

Because the CHO-K1 cells do not express endogenous CBS at significant levels, they are a suitable system for studying CBS mutants. Two 75-cm^2^ flasks containing Chinese hamster ovary–derived cells that express the reverse tetracycline-controlled transactivator (BD Biosciences, San Jose, California) were transiently transfected with pTRE-2hyg plasmid bearing either the wild-type or mutant human CBS sequences with Lipofectamine-2000 (Invitrogen, Carlsbad, California). The CBS expression was induced 4 hours after transfection with doxycycline (final concentration 200 ng/mL). About 20 hours after transfection the cells were harvested mechanically and pooled, cell extracts were prepared by osmotic lysis with phosphate buffer 30 mmol/L with PEE-W1 detergent,^17^ and protein concentration was determined as described above. All expressions were done in triplicate, and means are shown in the article. For kinetic studies large-scale expression was carried out, and higher variability in the amounts of mutant tetramers in lysates was observed (data not shown).

### CBS Activity and Kinetics Measurement

The CBS activity was assayed at homocysteine 10 mmol/L and serine 10 mmol/L by the previously published procedure^15^ with incubation at 37° C for 1 hour for *E. coli* lysates and 4 hours for CHO lysates with the following modification: mixture of unlabeled and ^14^C-labeled serine was replaced by ^2^H-labeled serine 10 mmol/L (Cambridge Isotope Laboratories, Andover, Massachusetts) and the amount of ^2^H-labeled cystathionine produced was determined by LC-MS-MS with a commercially available kit for amino acid analysis (EZ:faast; Phenomenex, Torrance, California) (Krijt et al, unpublished data).

The kinetic studies were performed in CHO lysates (approximately 30 to 40 μg of total cellular protein in 50 μL reaction) incubated for 30 minutes at 37° C. The above-described assay procedure was used maintaining one substrate at saturating concentration (40 mmol/L and 20 mmol/L of homocysteine and ^2^H-labeled serine, respectively) while using the concentration of the other substrate of 0.31, 0.62, 1.25, 2.5, 5, 7.5, 10, 12.5, 15, 20, 30, and 40 mmol/L. The K_m_ and V_max_ were calculated with KaleidaGraph software (Synergy Software, Reading, Pennsylvania).

### Western Blot Analysis

For Western blot analysis, cell lysates containing total protein 10 μg were electrophoresed on 4% to 15% gradient native polyacrylamide gels (BioRad precast gels) with Laemmli buffer system with or without SDS for SDS-PAGE and native-PAGE gel, respectively. The separated proteins were transferred onto polyvinylidene difluoride membrane (Immobilon-P; Millipore, Billerica, Massachusetts) with semi-dry blotting transfer technique. After transfer and overnight blocking of nonspecific binding sites with 5% non-fat dry milk, the membrane was incubated with immunopurified anti-CBS antibody diluted 1:5000 in 3% bovine serum albumin in 1 × phosphate-buffered saline solution (PBS) for 1 hour. After a series of washes the membrane was subsequently incubated with secondary anti-rabbit IgG antibody conjugated with HRP for 30 minutes (Pierce, Rockford, Illinois); the secondary antibody was diluted 1:30 000 in 5% non-fat dry milk dissolved in 1 × PBS/0.2% Tween 20. After a second series of washes the signal was visualized using the West Pico Super Signal system (Pierce Biotechnology, Rockford, Illinois) followed by bioimaging system ChemiGenius-Q (Syngene Inc, Frederick, Maryland) with cooled CCD camera. The amount of tetramers and high-molecular weight aggregates were quantified with Gene Tools software (Syngene Inc).

### Statistical Methods

Expected birth prevalence was calculated on the basis of Hardy-Weinberg equilibrium with a model published previously^10^ with added frequency of the c.1105C>T alleles. Confidence intervals of estimates were calculated with S-plus (Tibco Software, Inc, Palo Alto, California) and R software.

## Results

### Frequency of the c.1105C>T Mutation and Expected Birth Prevalence of Homocystinuria

We detected six c.1105C>T heterozygotes among 600 unselected Czech newborns; 1 individual was present in the Bohemian cohort and 5 in the Moravian cohort, respectively. The observed allelic frequency of c.1105C>T is 0.005 (95%CI 0.0018-0.011), which is similar to that observed in Norway.^12^ Assuming Hardy-Weinberg equilibrium the expected birth prevalence of homozygotes for this mutation is 1:40 000 (95%CI 1:8000-1:295 000). We have previously estimated incidence of homocystinuria in the Czech Republic with a composite model.^10^ In the model we have combined direct detection of 2 mutant alleles in unselected newborn blood samples with an inferred population frequency of 8 additional mutant alleles calculated from their number in patients who were ascertained by biochemical screening during a 20-year period. After adding the data on c.1105C>T from this study into the above-mentioned composite model, the expected birth prevalence of homocystinuria in the Czech Republic increased to 1:15 500 newborns (95% CI 1:7500-1:38 000).

### In Silico Analyses of the p.R369C Mutant

Arginine 369 is located in the beta strand 11 at a dimer-dimer interface of the CBS enzyme.^18^ In their 3-dimensional model of full-length CBS, Yamanishi et al^19^ predicted that this mutation abolishes an ionic interaction with D506 residue located in the carboxyterminal regulatory CBS domain, suggesting possible destabilization of tertiary structure. Analysis with Clustal-W and a series of CBS orthologs obtained from NCBI database showed that arginine 369 is highly conserved in mammals, and in other organisms, this positively charged amino acid may be replaced by positively charged lysine or uncharged tyrosine, valine, leucine, methionine or asparagine residues but not by cysteine (data not shown). This imperfect conservation of arginine in position 369 of the CBS polypeptide during evolution suggests that this amino acid residue is not absolutely critical for cystathionine beta-synthase function. Taken together these computer-simulated data suggest that the effect of this mutation on enzyme function may be rather mild.

### Properties of p.R369C Mutant Expressed in *E. coli*

The p.R369C mutant expressed at 37° C was present in bacterial extracts in amounts somewhat lower than the wild-type enzyme (66 % of CBS signal relative to the wild-type upon analysis by denaturing SDS-PAGE/western blotting; data not shown). Most of these mutant enzyme molecules misfolded, as demonstrated by the presence of only 8% of correctly folded tetrameric fraction of the p.R369C relative to the wild-type CBS enzyme when non-denaturing conditions for PAGE/Western blotting were used and data were normalized for CBS abundance (Figure). The relative activity—that is, specific activity normalized for the abundance of tetramers—of the p.R369C mutant was decreased to 34% of the wild-type (Table II).

It was shown previously^20^ that lower expression temperature may facilitate the attainment of correct tertiary and quaternary structure of misfolding-prone mutants. Indeed, folding and assembly of mutant p.R369C polypeptide chains expressed at 18° C improved substantially as the bacterial extracts contained 23% of tetramer compared with wild-type and the relative activity simultaneously increased to 67% (Table II). To examine whether the p.R369C mutation may alter the response to the natural allosteric activator of CBS—S-adenosylmethionine (SAM)—we measured the catalytic activity of the mutant expressed at 18° C in the absence and presence of 1 mmol/L SAM. The activity of the mutant increased 4.4 times in the presence of SAM, which is similar to the increase of 5.7 for the wild-type enzyme; these data demonstrate that the mutant enzyme retains its normal allosteric activation by S-adenosylmethionine.

The above data show that the p.R369C CBS mutant is intrinsically prone to misfolding with simultaneous loss of activity, and that this property may be reversed by folding permissive conditions. Moreover, the expression studies demonstrated that even the correctly folded and assembled tetramers exhibit only 34% to 67% relative activity per tetramer amount compared with the wild-type enzyme. It was shown previously that despite their limitations the prokaryotic expression systems permit examination of intrinsic properties of mutant enzymes such as cytosolic phenylalanine hydroxylase, and expression in mammalian systems mimics more faithfully the in vivo situation in human beings.^21^ Therefore we subsequently tested the properties of the mutant enzyme in a mammalian system.

### Properties of p.R39C Mutant Expressed in CHO-K1 Cells

The p.R369C mutant expressed in mammalian cells was present in amounts similar to the wild-type enzyme (105% of the wild-type enzyme when assessed by Western blotting under denaturing conditions, data not shown). Surprisingly, the amounts of correctly folded tetramers and higher order oligomers were slightly increased to 129% compared with wild-type enzyme after correction for the CBS abundance. However, the relative activity of these correctly folded mutant oligomers was decreased to 54% of the wild-type tetramers (Table II). Taken together the net effect of the p.R369C substitution on quaternary structure and activity appears to be moderate in mammalian cells.

All experiments showed that despite varying extent of correct folding in either the mammalian or prokaryotic system the relative activity of the p.R369C mutant is similarly decreased to about half of the wild-type enzyme. Such behavior of the p.R369C mutant is expected to result from altered kinetic properties. Therefore we determined the Michaelis constant K_m_ for both substrates and we measured the V_max_ of reaction in crude CHO cell extracts. The p.R369C mutant exhibited K_m_ for serine and homocysteine similar to K_m_ of the wild-type CBS (Table II). In contrast the V_max_ of the mutant was reduced to 29% of the wild-type enzyme and to 55% when corrected for the abundance of tetramers in samples that were used for kinetic studies. Considering the limitations of analyses in crude extracts all these data suggest that the p.R369C mutant does not have lower affinity for its 2 substrates but that the velocity of the reaction is approximately halved.

## Discussion

The surprisingly high frequency of the c.1105C>T allele among Norwegian newborns suggests that this *CBS* mutation may be the most common cause of homocystinuria in North Europeans.^12^ However, functional studies indicated that it may only be a frequent neutral polymorphism, although the authors discussed a possibility that the yeast may not model this mutation properly.^14^ Because detailed functional studies and data on frequency in other populations have been lacking, we aimed in our study at exploring the properties of p.R369C mutant enzyme and at determining the prevalence of the c.1105C>T allele in another European population.

From clinical standpoint the question whether the c.1105C>T mutation causes homocystinuria or whether it is only a functionally neutral genetic variant is of key importance. Three lines of evidence support the hypothesis that it is indeed pathogenic. First, this mutation was found at 1 parental allele in 2 independent patients with CBS deficiency (Table I; patients 1 and 2), demonstrating that the p.R369C mutant enzyme produced from the c.1105C>T allele has impaired catalytic activity because it is not able to functionally complement the mutant protein synthesized from the other parental allele. Second, the expression studies presented in this article strongly suggest that the mutant enzyme is intrinsically prone to misfolding but more folding permissive conditions of mammalian cells enable formation of normal amounts of tetramers; in human beings carrying this mutation the tendency to misfold may only manifest under conditions that disfavor folding. Third, regardless of the expression system, the relative activity of p.R369C mutant normalized for tetramer abundance is decreased to about one half of the wild-type enzyme. We have shown that this low activity is not caused by altered binding of substrates but rather by a decreased velocity of the enzymatic reaction.

The properties of mutant enzyme expressed in cells, however, cannot unequivocally answer the question whether the p.R369C mutation is pathogenic in vivo. The evidence has to come from clinical observations; however, there is a scarcity of reports on CBS-deficient patients carrying the c.1105 C>T variant. This discrepancy between the numbers of expected and reported homozygotes/compound heterozygotes for c.1105 C>T may be due to several reasons, for example, (1) patients may die antenatally, (2) patients may have a phenotypically different disease, which does not resemble homocystinuria (such an example is the mutation p.V377I in mevalonate kinase gene^22^), (3) the disease manifests in an unrecognized mild adult-onset form, or (4) patients do not have any clinical symptoms at all. The late onset of symptoms, lack of mental retardation, and of connective tissue involvement, and pyridoxine responsiveness in the known patients carrying the p.R369C mutation favor the latter 2 hypotheses of a mild to null phenotype, which possibly escapes diagnosis. It is puzzling that such individuals are not being reported among patients with moderate to severe hyperhomocysteinemia who had been ascertained during a thrombophilia workout. In summary, it is conceivable that in human beings the p.R369C mutation is not functionally neutral in vivo although severity and spectrum of clinical consequences is at present unknown; only future reports on phenotypic features in homozygotes and additional compound heterozygotes carrying this mutation will resolve this issue.

The second aim of our study was to determine the frequency of c.1105 C>T in another European population. The 0.5% frequency of c.1105 C>T alleles in predominantly Slavic population of the Czech Republic is similar to 0.8% frequency determined by Refsum et al^12^ in Norwegian newborns or 0.5% shown for 200 North American adult control subjects by Kim et al.^14^ Although data from other European populations are lacking, similar frequencies in unrelated Norwegians, Czechs, and North Americans suggest that this variant allele may be of ancient origin and that it may be common in populations of European descent. This high population frequency of mutant CBS alleles may have important consequences for newborn screening. The expected frequency of homocystinuria because of 6 mutations in Norway^12^ and 11 mutations in the Czech Republic (this study) are similarly high, being 1:6400 and 1:15 500, respectively. Provided that p.R369C is indeed pathogenic, the expected frequency of homocystinuria in 2 European populations is similar to the frequency of other common inborn errors of metabolism such as phenylketonuria or MCAD deficiency.^7^ Such frequency and encouraging efficacy of therapeutic interventions in early detected patients with CBS deficiency (summarized in reference 2) rank thus CBS deficiency among suitable candidates for an efficient newborn screening.

Unfortunately, the existing newborn screening programs for CBS deficiency are based on detecting moderately to grossly elevated methionine concentrations by various techniques, including tandem mass spectrometry. It has been shown that patients may not be ascertained if higher cut-off methionine levels are used.^23^ Especially the pyridoxine-responsive forms, which are present in at least 50% of patients with homocystinuria,^1^ seem to escape diagnosis by newborn screening programs.^7^ This is demonstrated by only a single patient with pyridoxine-responsive homocystinuria detected among 36 Irish and U.S. patients that were ascertained in the newborn period.^8,23^ Total homocysteine measurement in blood samples^24^ or dried blood spots^25^ may be a more reliable approach to detecting neonates with homocystinuria. Unfortunately, total homocysteine is not routinely analyzed in newborn screening programs because of the necessity of releasing homocysteine from its bond to plasma proteins in an additional pre-derivatization reduction step. Because cystathionine is decreased and methionine normal to increased in CBS deficiency, the addition of a cystathionine assay with calculation of cystathionine-to-methionine ratio may be an approach for increasing the number of detected presymptomatic newborn patients with homocystinuria. The molecular epidemiology data reported previously^12,14,26^ and in this study challenge our perception of homocystinuria as a rare disease and should stimulate a discussion on how to increase the efficacy of newborn screening for homocystinuria in populations of European origin.

## Figures and Tables

**Figure:**
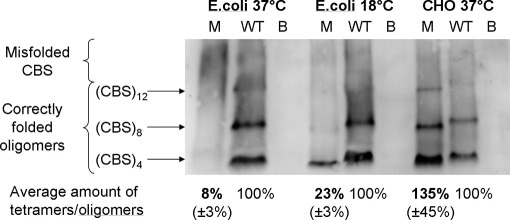
Quaternary structure of p.R369C mutant under different expression conditions. The p.R369C mutant was expressed in both *E. coli* and CHO cell-based systems, crude extracts were electrophoresed in gradient polyacrylamide gels under non-denaturing conditions followed by Western blotting (see Methods for details). A representative gel is shown, the sharply demarcated fractions (CBS)_4_, (CBS)_8_, or (CBS)_12_ contain 4, 8, or 12 CBS subunits, respectively; the smear in the high molecular weight fraction contains misfolded CBS. The individual lanes contain either the p.R369C mutant *(M)*, wild-type CBS *(WT)*, or blank *(B*, i.e. either *E. coli* transformed with empty vector or CHO cells transfected with empty vector pTRE2hyg, respectively). The line *“Average amount of tetramers/oligomers”* shows the relative amounts of the sum of correctly folded mutant tetramers and oligomers relative to the wild-type CBS enzyme, data are means of 3 independent expressions with SD in parentheses.

**Table I:** Known patients with a CBS deficiency carrying the c.1105C>T (p.R369C) mutation

Ancestry	Genotype⁎	Disease severity	Vitamin B_6_†	Comments	Reference
Norwegian-sib pair	Allele 1: p.R369C Allele 2: p.I278T	variable among siblings	+	A sibpair diagnosed at 33 and 27 years of age, respectively. The older sister was symptom free, the younger brother had severe psychiatric disease. Plasma tHcy concentrations in these 2 patients were 245 and 130 μmol/L before, and 8 and 18 μmol/L after pyridoxine administration (40 mg/d), respectively.	14
Anglo-Celtic	Allele 1: p.R369C Allele 2: c.533del18	mild	+	The patient manifested by pulmonary embolus at the age of 23 years after the birth of her son. She was ascertained by family screening at 24 years of age since her son, who inherited from her the 533del18 mutant allele, was diagnosed with CBS deficiency.	4
Dutch	Both alleles: [p.R369C; p.R491C]	severe	+	Severely affected patient with ectopic lenses, other connective tissue abnormalities, thromboembolism and psychosis but without mental retardation, age of diagnosis is not given. The relative contribution of the 2 mutations linked in *cis* to the severe inactivation of CBS enzyme has not been determined.	13

⁎ Genotypes of both alleles are shown with description of either cDNA nucleotide change (signified by “c.”) or of the predicted amino acid substitution (signified by “p.”).

† This column shows pyridoxine responsiveness; in references 4 and 11 the vitamin B_6_ responsiveness is defined as decrease of plasma tHcy below 50 or 60 μmol/L after pyridoxine treatment, respectively.

**Table II:** Catalytic activity and kinetic properties of the p.R369C mutant

	Wild-type CBS	p.R369C mutant
Specific activity in crude extracts (nmol cystathionine/mg total cellular protein/hour)⁎		
*E. coli* 37° C	142.7 ± 27.2	2.8 ± 2.2
*E. coli* 18° C	85.5 ± 27.0	13.1 ± 0.4
CHO-K1 37° C	111.1 ± 39.7	71.2 ± 27.3
Relative activity in crude extracts (specific activity corrected for tetramer abundance, in % of wild-type enzyme)		
*E. coli* 37° C	100%	34% ± 26%
*E. coli* 18° C	100%	67% ± 8%
CHO-K1 37° C	100%	54% ± 24%
Kinetic properties of wild-type and p.R369C mutant enzyme in crude extracts after expression in CHO-K1 cells†		
K_m_ serine (mmol/L)	10.1	8.1
K_m_ homocysteine (mmol/L)	5.8	7.5
V_max_ (nmol/h/mg total cellular protein/)	1715	500
Normalized V_max_ (nmol/h/arbitrary units of CBS tetramer)	100%	55%

⁎ Numbers shown are means of 3 independent expression experiments with SD.

† Numbers shown are means of 2 independent experiments (with the exception of K_m_ for homocysteine, which was determined only once).
